# Small Bowel Intussusception Due to Rare Cardiac Intimal Sarcoma Metastasis: A Case Report

**DOI:** 10.3389/fsurg.2021.743858

**Published:** 2021-10-04

**Authors:** Marco Chiarelli, Mauro Zago, Fulvio Tagliabue, Morena Burati, Cristina Riva, Alice Vanzati, Emanuele Dainese, Francesco Gabrielli, Angelo Guttadauro, Matilde De Simone, Ugo Cioffi

**Affiliations:** ^1^Department of Emergency and Robotic Surgery, A. Manzoni Hospital, ASST Lecco, Lecco, Italy; ^2^Department of Pathology, A. Manzoni Hospital, ASST Lecco, Lecco, Italy; ^3^Department of Surgery, Istituti Clinici Zucchi, University of Milan Bicocca, Monza, Italy; ^4^Department of Surgery, University of Milan, Milano, Italy

**Keywords:** cardiac tumor, intimal sarcoma, metastasis, intussusception, bowel obstruction

## Abstract

**Background:** Intimal sarcomas are rare malignant mesenchymal tumors arising from the heart and large blood vessels. Their intraluminal growth leads to vascular obstructive symptoms and peripheral neoplastic embolization. Direct infiltration of the lungs or metastases to the pulmonary system, occur in 40% of cases and extrathoracic spread is frequent, also in presentation. Intussusception is an unusual event in adults, accounting for <5% of bowel obstructions. In most cases it is caused by a malignancy and requires surgical resection.

**Case Presentation:** We describe a rare case of a 50-year-old man suffering of bowel obstruction due to intussusception sustained by a small bowel metastasis of a primary cardiac intimal sarcoma. One year and a half before the onset of abdominal symptoms, a grade II intimal sarcoma was removed from his left atrium and consequently he followed a chemotherapy protocol. Four months later a CT scan revealed local recurrence. Eighteen months after heart surgery he referred to the ER with abdominal pain. CT scan showed an ileal intussusception and the patient was scheduled for surgery. A tract of 10 cm ileus was removed containing an intramural polypoid solid mass. Histological analyses revealed a grade II intimal sarcoma consistent with his first diagnosis.

**Conclusion:** Primary heart tumors are late found and often partially resected, therefore metastatic pathways are to be expected. Adult small bowel intussusception is a rare event and caused by a malignancy in one third of cases. Therefore, our recommendation is to always resect the tract involved in order to perform a proper diagnosis.

## Introduction

Primary cardiac sarcomas are extremely rare neoplasms and show a malignant behavior in 25% of cases (1). In the past the primary cardiac intimal sarcoma (PCIS) was thought to be extremely rare and infrequently diagnosed. On the contrary, a recent retrospective morphological and molecular study demonstrated that PCIS represents the most frequent cardiac sarcoma accounting for 42% of cases, followed by angiosarcoma and undifferentiated sarcoma, respectively 26 and 22% of them (2). Intimal sarcomas are malignant mesenchymal tumors typically arising from heart and large blood vessels of the systemic or pulmonary circulation (3). They are characterized by poor prognosis and frequent local recurrence, since their complete surgical resection is often difficult to achieve. Distant metastases, mainly in the form of intravascular embolization, occur in the majority of the patients and in 40% of the cases are found in the lungs. Several other metastatic sites have been reported including pancreas, kidney, brain, lymph node and skin (4, 5). Intussusception is a rare event in adults leading to <5% of bowel obstruction. In most cases it is caused by a malignancy and requires surgical resection (6).

In this paper, we report an extremely rare case of small bowel intussusception sustained by an intramural polypoid mass, proven to be a metastasis from a PCIS.

## Case Presentation

### Chief Complaints

A 50-year-old Caucasian male patient presented to our Emergency Room (ER) with nausea and abdominal pain.

### History of Present Illness

At ER admission, he referred a 3-week history of recurrent abdominal pain. He also referred one episode of vomit and constipation from 2 days.

### History of Past Illness

The recent patient history included cardiac surgery 18 months before, aimed to remove a left atrium polylobate neoplastic mass, measuring ~8 cm, partially infiltrating pulmonary veins and the aortic valve. Self-heart transplant and aortic valve plasty were performed, but the ablation resulted incomplete (R1, resection margins were microscopically positive for tumor cells). Pathology examination revealed a moderately-poorly differentiated intimal sarcoma, characterized by proliferation of atypical spindle and pleomorphic cells, with scattered, focally brisk mitotic figures and spotty necrosis. Proliferation index, measured by Ki-67, was 25–30%. Immunophenotyping showed positivity for actin, desmin, myosin, vimentin and MDM2 antibodies; no reaction was observed for the antibodies S100, ALK 1 and CD34. MDM2 gene amplification was observed by FISH analysis. After surgery, CT and PET scan, performed for staging purpose, did not show metastatic lesions. The patient underwent two cycles of chemotherapy with Doxorubicin (75 mg/mq) and Isophosphamide (5,000 mg/mq). Four months after surgery, CT scan showed an atrial mass consistent with local recurrence. Oncologic evaluation suggested continuing another cycle of chemotherapy, but it was suspended due to patient's intolerance. Nevertheless, patient accepted to undergo a cycle of 30 fractions of mediastinal radiotherapy (50 Gy + 10 Gy boost). Cardiac ultrasounds performed during follow-up showed no progression of the heart recurrence mass.

*Physical examination* Abdomen was flat and soft and did not show signs of rebound tenderness, no masses were palpable and peristalsis was present.

### Laboratory Examinations

Blood test showed mild leukocytosis 11 × 10^9^/L, with normal hematocrit and platelet count. Hemoglobin was 14 g/dL. No other alterations were registered, included C-reactive protein which resulted negative (0.36 mg/dL).

### Imaging Examinations

**Figure 1:**
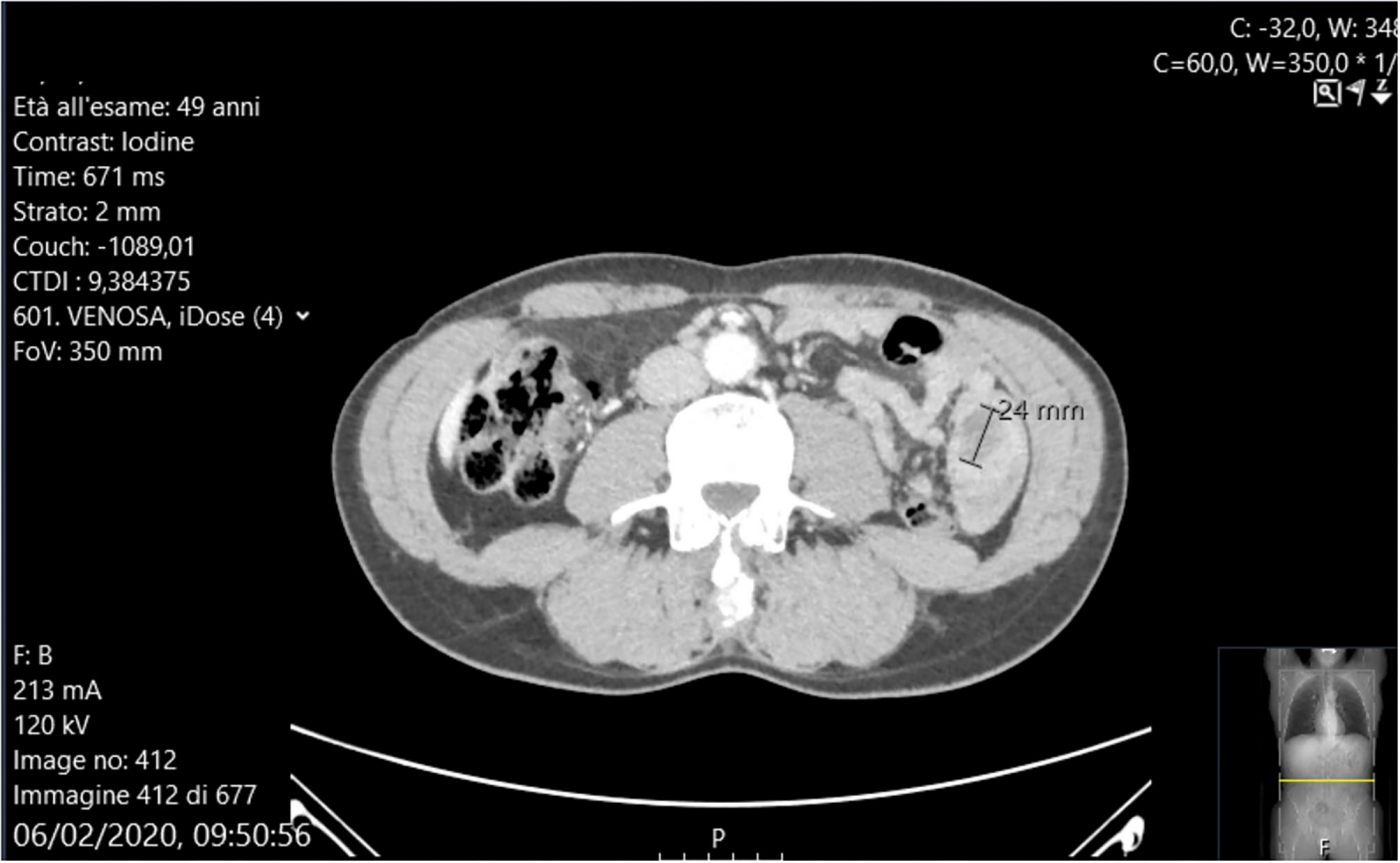
Ct-scan showing the invaginated tract containing a 24 mm mass.

Abdominal X-rays were normal. A CT scan showed moderate small bowel distention caused by an ileal intussusception containing a 24 mm mild hypodense lesion (Figure 1).

### Surgical Treatment

**Figure 2:**
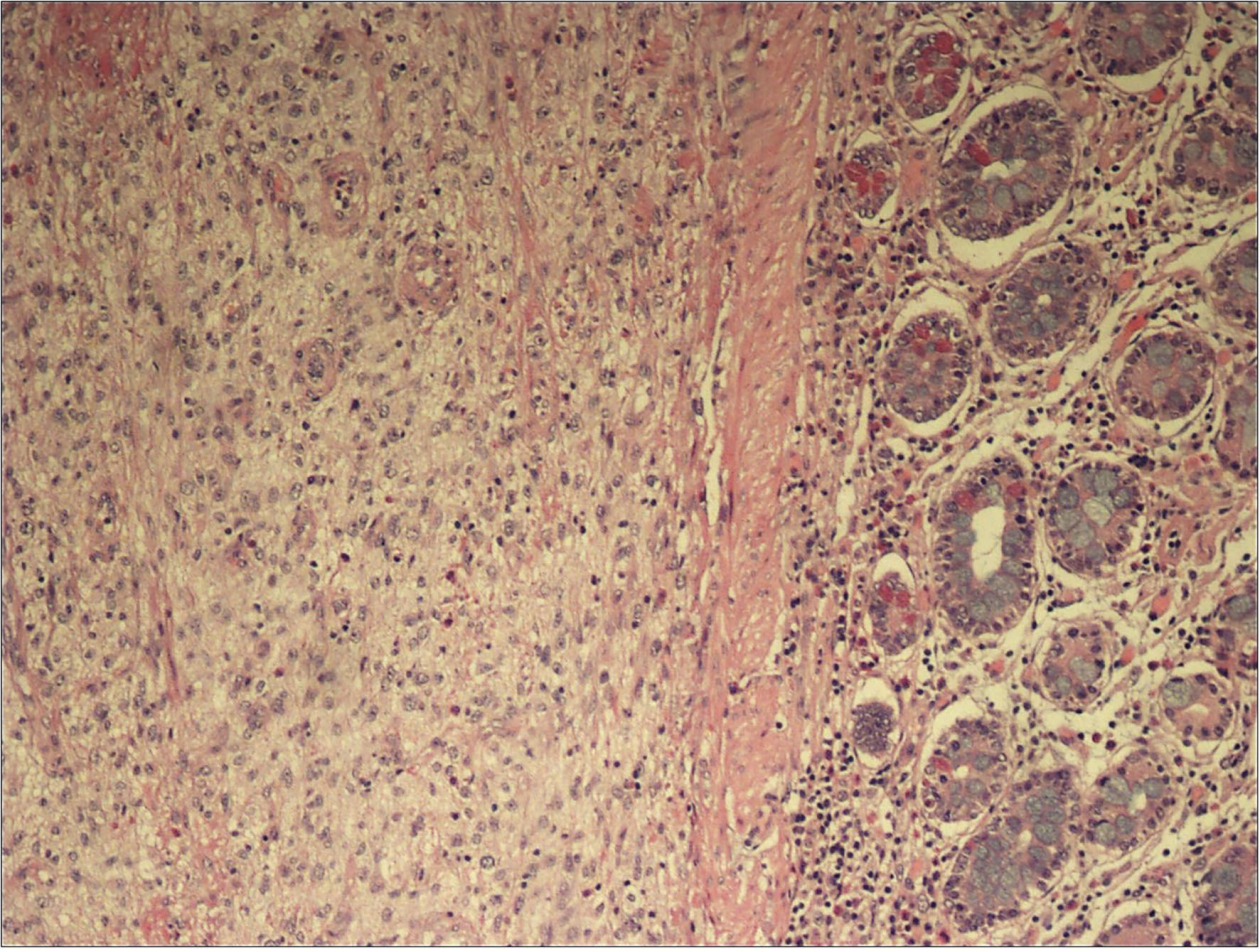
H&E, 100x magnification: Ileal submucosal spindle cell proliferation.

**Figure 3:**
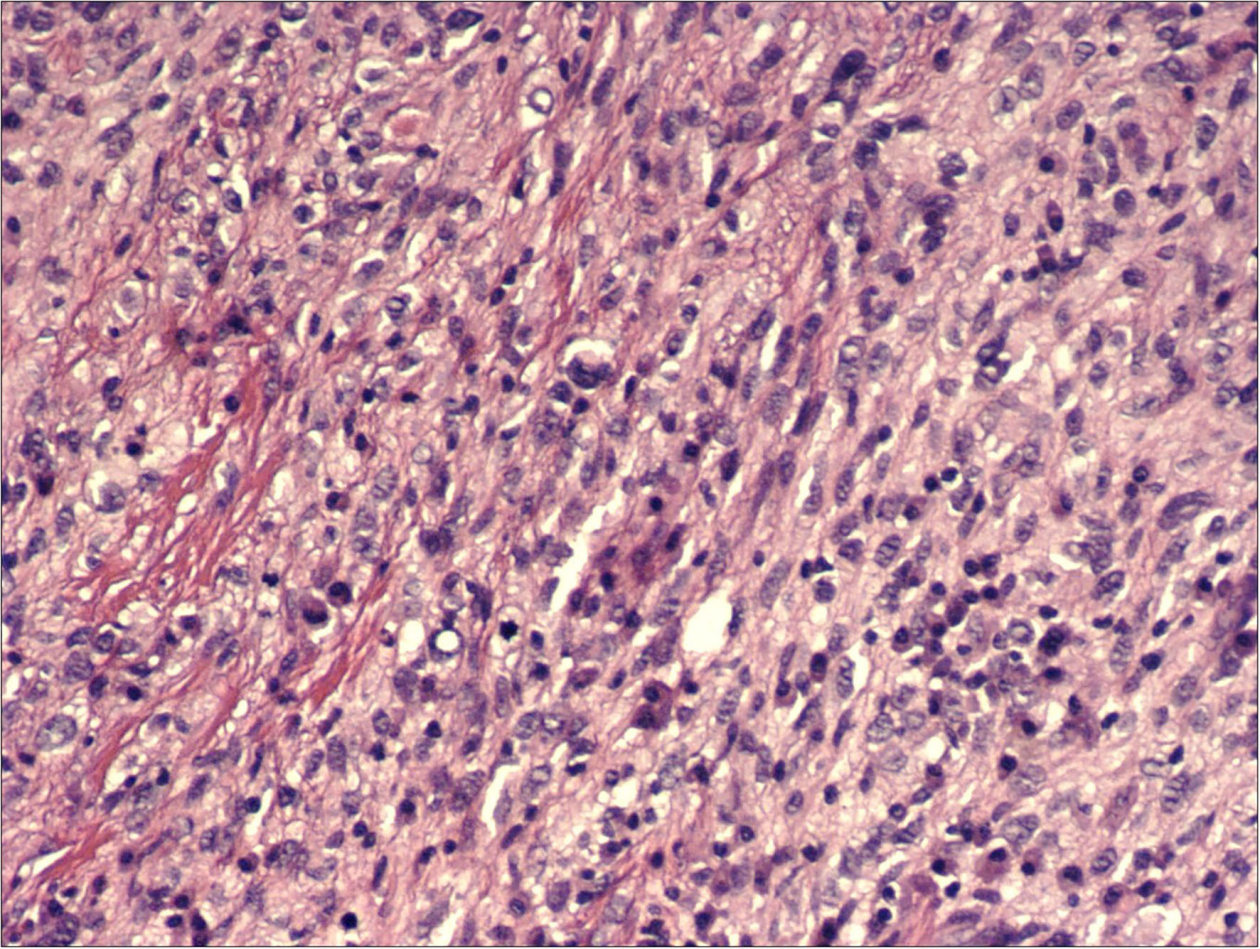
H&E, 200x: hypercellular proliferation composed of spindle cells with nuclear atypia and intermingled moderate amount of inflammatory cells.

**Figure 4:**
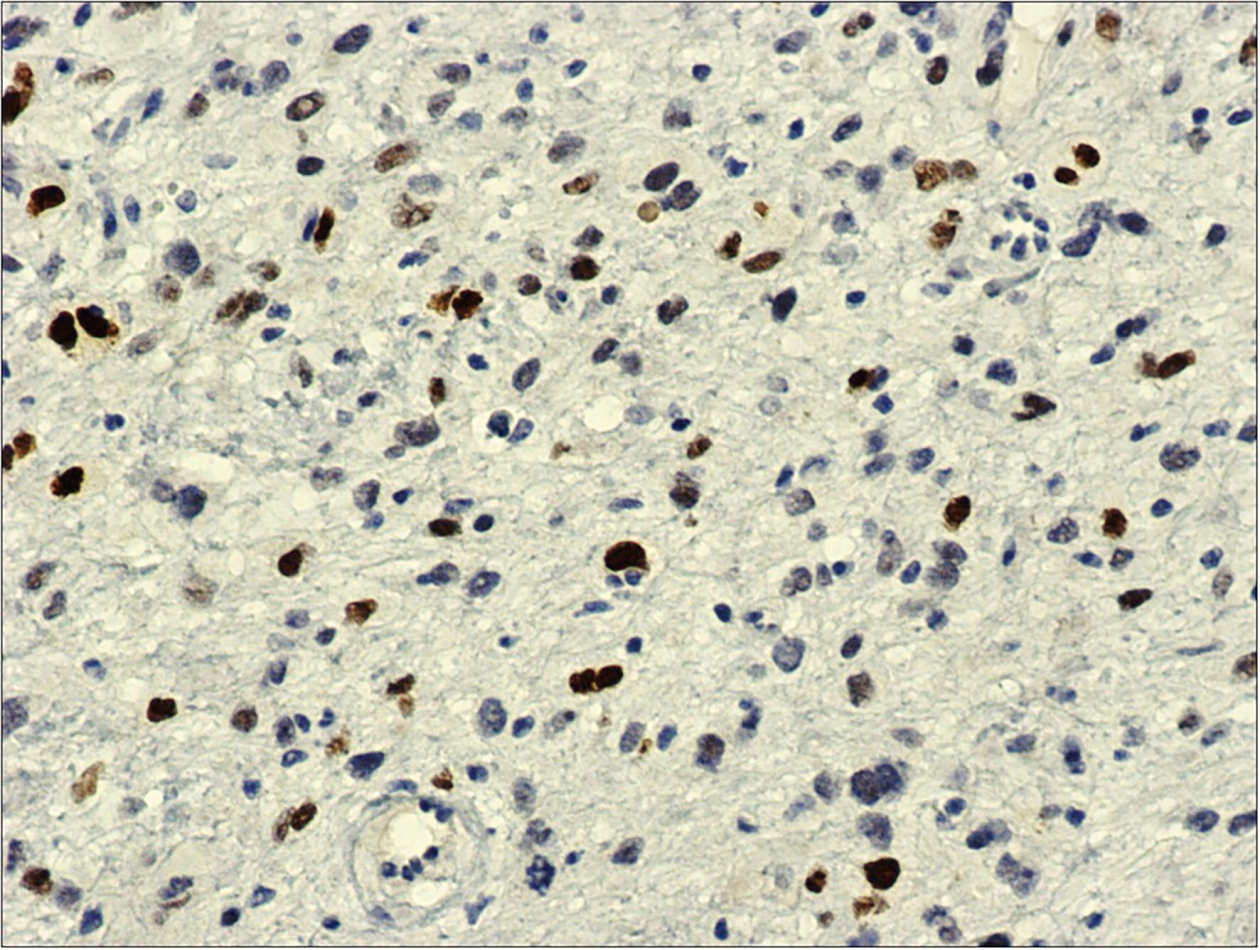
MDM2, 20x: immunohistochemistry against MDM2-antibody shows diffuse and intense nuclear reaction.

Patient underwent urgent laparotomy. The abdominal cavity was explored with identification of the invaginated mid-ileal tract, associated with proximal small bowel dilatation. Intussusception was manually reduced and a 2 cm lesion was palpable inside the lumen. A 10 cm-segmental ileal resection was performed, removing the tract containing the mass with its lymph nodes; consequently, digestive continuity was restored with a manual end to end anastomosis. Pathology examination revealed a malignant mesenchymal lesion composed by atypical spindle and pleomorphic cells with brisk mitotic activity; the immunoprofile was characterized by MDM2, and actine positivity (CD34, DOG1, and CD117 resulted negative). FISH analysis demonstrated MDM2 amplification. These findings overlapped the features of the previous cardiac neoplastic lesion, so that a diagnosis of intimal sarcoma metastatic to the ileal wall, with submucosal polipoid growth and mucosa ulceration, was made (Figures 2–4).

### Outcome and Follow-Up

Recovery was uneventful. Oral feeding was resumed 3 days after surgery, and he was discharged on 5th post-operative day. He started a radiotherapy protocol with administration of 60 Gy of radiation dosage. A chemotherapy schedule based on Doxorubicin plus Isophosphamide was started. Nevertheless, due to high toxicity and low compliance the patient underwent no other chemotherapy cycles, preferring a very strict follow-up. At present, further follow-up exams always showed negative.

All diagnosis and decisions were discussed with the patient by a multi-disciplinary team, including oncologists, surgeons, cardiac surgeons and radiotherapists. The patient gave consent to both cardiac and bowel surgery and accepted radiotherapy and chemotherapy. He was also informed of the uniqueness of the case and he consented on publishing it as case report in medical literature.

## Discussion

Primary cardiac neoplasms are extremely rare tumors. Their incidence ranges between 0.001 and 0.03%. Approximately 95% of them are soft tissue tumors, most often sarcomas, characterized by an aggressive behavior (1). The prognosis of these neoplasms is poor and it is more influenced by surgical radicality than by histologic subtype. A recent retrospective study describing immunohistochemical, molecular, and clinico-pathological features of the largest series of primary cardiac sarcomas (2) demonstrated intimal sarcoma, previously never reported in the heart, as the most common entity (3), accounting for 42% of the cases. Angiosarcomas and undifferentiated sarcomas account respectively for 26 and 22% of the cases. Synovial sarcomas and leiomyosarcoma are much more infrequent (3). Intimal sarcoma is more commonly encountered in the large arterial blood vessels including pulmonary artery and aorta (7, 8). Cardiac intimal sarcoma is usually left atrium located, while angiosarcoma and spindle cell pleomorphic tumors are right sided (9–11). The mean age is 50 years, with a female/male ratio 1.8:1. Mean tumor size is about 60 mm, and intimal sarcoma can grossly appear as a polypoid mass, often mistaken for myxoma on imaging, especially for left atrium site (2, 12).

Histologically, intimal sarcomas are usually poorly differentiated high grade malignant mesenchymal tumors characterized by hypercellular proliferation of atypical spindle cells with variable degrees of atypia, brisk mitotic activity, necrosis, and nuclear polymorphism (13). Prominent spindling and bundling can mimic leiomyosarcoma. angiosarcoma, synovial sarcoma, myxofibrosarcoma, dedifferentiated liposarcoma and secondary tumors, such as melanoma and carcinoma, must be rule out. Approximately all cases of intimal sarcoma show positive immunoreactivity for vimentin and MDM2; often also alpha-actin and desmin are positive. On the contrary CD34, CD117, DOG1, MART-1, S-100 and cytokeratins are negative. In the majority of the cases MDM2 amplification in 12q13 is detected by FISH and it is desirable to diagnosis purposes (2). Interestingly, the amplification pattern in intimal sarcoma is quite different from that usually seen in dedifferentiated liposarcoma, being organized in small clusters scattered throughout the cell, instead of large clusters (3).

Clinical presentation of a cardiac intimal sarcoma is largely non-specific. Tumor growth can lead to congestive heart failure, dyspnea and embolism. The onset is often late: therefore, systemic embolization, conduction abnormalities, pericardial effusion and metastases (20%) can already be present (12). Metastatic pulmonary spread frequently occurs (40%). Several other metastatic sites have been reported including pancreas, kidney, brain, lymph node and skin (4, 5).

Intimal sarcoma of the heart is related to a poor prognosis. Mean survival is 3–17 months (2, 10). The therapy is based on surgery, chemo- and radiotherapy. Complete resection, difficult to achieve, is an important predictor of DFS11 and, unfortunately, 1-year recurrence is frequently reported. Chemotherapy based on Isophosphamide-Epirubicin (or Doxorubicin) and Cyclophosphamide, Vincristine, Dacarbazine (CyVADIC) combination commonly used on soft tissue sarcomas (14) was proven to improve survival.

Intestinal invagination consists in the telescoping of a proximal segment of the bowel within the lumen of the adjacent segment. The condition is rare in adults, while it is frequently seen in pediatric population. Adult intussusception (AI) accounts for only 5% of cases, mostly sustained by a lead point that can be a benign lesion or a malignancy (responsible of 30% of all small intestine invagination) (6, 15). An enormous variety of lesions have been described in international case reports as the cause of AI: small bowel benign neoplasm, gastro-intestinal stromal tumor (GIST), intestinal adenocarcinoma, Meckel's diverticulum, appendix, Peutz Jeghers syndrome, Crohn's disease, intestinal lymphoma, coeliac disease and congenital adhesions (16–22). Metastatic tumor being the cause on AI is often described. In most cases, the primitive cancer is a melanoma but also sarcomas, renal, lung and esophageal cancers are described. As easily predictable, the nature of the mass responsible of the invagination, strongly affects patients' survival and therapeutic strategy (23). Moreover, when a malignant lesion causing AI is found, synchronous lesions must be searched, looking for the primitive disease.

CT scan is the pivotal examination for the diagnosis of AI; nevertheless, in the case of finding an intestinal mass, it is very difficult to characterize the tumor. Moreover, at CT scan imaging, intimal sarcoma can be misdiagnosed as a GIST. In these cases, immunohistochemical analysis is crucial to rule out the difference. GISTs usually stain positive for CD34 and, in ~88% of cases, for both CD117 and DOG-1. In a recent study, a positive expression of CD117 and DOG-1 in gastrointestinal stromal tumors was shown in 95.71 and 88.57% of cases respectively (24). Malignant melanoma cells stain positively with antibodies to vimentin, Human Melanoma Black 45 (HMB45), Melan A (MART1) and S-100 protein (25), negative in our case. Another fundamental marker to be cited is MDM2 amplification. MDM2 is a negative regulator of p53 and it is tipically amplified in tumors such as well-differentiated liposarcoma, atypical lipomatous tumor, intimal sarcoma, and low-grade osteosarcoma.

AI is responsible of 1% of all bowel obstructions (26) and its diagnosis can be somehow challenging: patients often refer long history of recurrent abdominal pain and the initial diagnosis often is missed or delayed. CT scan is often the choice exam to investigate prolonged abdominal pain. CT scan showing an inhomogeneous soft-tissue mass that was target- or sausage-shaped has been proved to be superior to other diagnostic modalities with better accuracy in preoperative diagnosis (27). Moreover CT can detect vascular compromise, and can be used to predict the likelihood of self-resolution (28, 29). The optimal management of AI is controversial, and there is no universal accordance on that. In front of a diagnosis of intestinal intussusception the surgeon must decide whether to wait and see or to operate. Even if there are no international guidelines, in literature, a cautious approach of conservative management can be a valid option in young patients (low risk of malignancy) who present with a vague history of abdominal symptoms suggestive of obstruction and a short segment intussusception (30). Of interest, a recent study, has demonstrate that intestinal invaginations involving <3.5 cm of bowel are likely to spontaneously reduce and could be worthy of a conservative approach (28). In all other cases, especially in case of acute abdomen with CT signs of vascular sufferance, surgery must be considered the principal treatment strategy.

The main surgical strategies are primary en-bloc resection vs. initial reduction. Both laparotomic and laparoscopic approaches are described, depending on the clinical condition of the patient, the extent of intussusception and the presence of an underlying disease (31). In most case series, a resection of the invaginated tract is performed, especially when a mass is palpable in the lumen (32). Manual reduction of the intussusception is rarely described (33), but it is advised, when possible (benign lesions) to reduce the extent of resection or to avoid the short bowel syndrome (34).

The reported case shows some interesting aspects. Intimal sarcoma is the most common entity among cardiac tumors, even if extremely rare. Some clinic-pathological features show similarities with literature data, including age, site and local relapse (35–37). In fact, the patient was a 50-year-old-man and presented with a left sided heart mass, which was incompletely removed leading to a local recurrence after 4 months. Nevertheless, symptomatic intramural small bowel metastasis appears exceedingly uncommon. Moreover, only 1% of all bowel obstructions are caused by small intestine invagination in the adult (38–40). As far as we know, there are no other publications in international literature describing an intussusception in an adult patient due to a small bowel localization of an heart intimal sarcoma.

## Conclusion

Two lessons may be learned from our case. First of all, diagnosis of heart tumors is often late, compromising complete ablative surgery and therefore facilitating an expected metastatic spread. Secondly, adult small bowel invagination is a rare event, sustained in one third of cases by a malignancy and it is advisable to resect the affected intestinal tract in order to perform proper diagnosis.

## Data Availability Statement

The original contributions presented in the study are included in the article/Supplementary Material, further inquiries can be directed to the corresponding author/s.

## Author Contributions

MC and MZ designed the study, analyzed the data, and revised the manuscript. FT designed the overall concept of the study and revised the manuscript. MB, MD, FG, and AG contributed to data acquisition and drafted the manuscript. CR, AV, and ED contributed to laboratory analyzes and draft the pathology section. UC contributed to the design the study and reviewed the discussion and conclusions. All authors approved of final the version of the manuscript to be published.

## Conflict of Interest

The authors declare that the research was conducted in the absence of any commercial or financial relationships that could be construed as a potential conflict of interest.

## Publisher's Note

All claims expressed in this article are solely those of the authors and do not necessarily represent those of their affiliated organizations, or those of the publisher, the editors and the reviewers. Any product that may be evaluated in this article, or claim that may be made by its manufacturer, is not guaranteed or endorsed by the publisher.
