# Supplementary material for: Small Bowel Intussusception Due to Rare Cardiac Intimal Sarcoma Metastasis: A Case Report
Source: Front Surg. 2021 Oct 4;8:743858. doi: 10.3389/fsurg.2021.743858 (PMC8521089; doi:10.3389/fsurg.2021.743858)
Supplement: Supplementary file 1 [file Data_Sheet_1.docx]

Still ongoing, negative

**Follow-up**

Weakness and signs of heart failure

Echocardiogram and CT angiogram: lesion in the left atrium, with large base of implant and partial occlusion of inferior pulmonary veins

Self heart-transplant with aortic valve plasty to remove a 8 cm polylobate neoplastic mass

Intimal heart sarcoma

CT - Doxorubicin (75 mg/mq) and Isophosphamide (5000 mg/mq)
+

Mediastinic RT (30 fr)

Abdominal pain

Moderate small bowel distention caused by an ileal intussusception

Negative

Intussusception caused by a 2 cm lesion that was palpable inside the lumen leading to a 10 cm-segmental ileal resection, removing the tract containing the mass with its lymph nodes

Intimal sarcoma metastatic to the ileal wall, with submucosal polipoid growth and mucosa ulceration

No CT due to toxicity and low compliance of the patient

but

RT with administration of 60 Gy of radiation dosage

**First ER admission**

**Imaging and exams**

**Heart surgery**

**Chemo- and Radio-thrapy**

**Follow-up**

**ER admission**

**Imaging and exams**

**Abdominal surgery**

**Pathology examination**

**Chemo- and Radio-therapy**

**Pathology
examination**

Two years later
